# Cation-induced speciation of port-size during mordenite zeolite synthesis†

**DOI:** 10.1039/d3ta03444e

**Published:** 2023-09-28

**Authors:** Sebastian Prodinger, Izar Capel Berdiell, Tomas Cordero-Lanzac, Odd Reidar Bygdnes, Bjørn Gading Solemsli, Karoline Kvande, Bjørnar Arstad, Pablo Beato, Unni Olsbye, Stian Svelle

**Affiliations:** a Center for Materials Science and Nanotechnology (SMN), Department of Chemistry, University of Oslo 1033 Blindern 0315 Oslo Norway sebastian.prodinger@smn.uio.no; b SINTEF Industry Forskningsveien 1 0373 Oslo Norway; c Topsøe A/S Haldor Topsøes Allé 1 2800 Kongens Lyngby Denmark

## Abstract

Mordenite (MOR) zeolite, an important industrial catalyst exists in two, isostructural variants defined by their port-size, small and large-port. Here we show for the first time how a systematic, single-parameter variation influences the synthesis out-come on the final MOR material leading to distinctly different catalysts. The cation identity has a direct impact on the synthesis mechanism with potassium cations generating the more constrained, small-port MOR variant compared to the large-port obtained with sodium cations. This was expressed by different degrees of accessibility ascertained with a combination of toluene breakthrough and temperature programmed desorption (TPD), propylamine TPD, as well as sterically sensitive isobutane conversion. Rietveld refinement of the X-ray diffractograms elucidated the preferential siting of the smaller sodium cations in the constricted 8-ring, from which differences in Al distribution follow. Note, there are no organic structure directing agents utilized in this synthesis pointing at the important role of inorganic structure directing agents (ISDA).

## Abstract 2

Varying the inorganic cation in the synthesis of MOR zeolite results in crystalline material of varying port-size, ascertained by gaseous uptake of toluene. Al siting is found to be the cause of this and differences on catalytic behavior follow.

## Graphical abstract

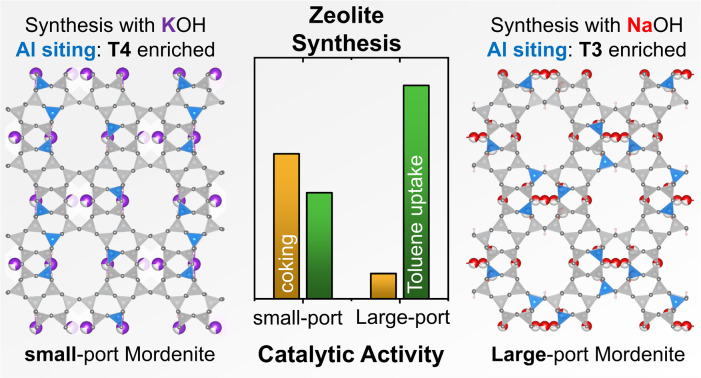

## 1. Introduction

Among zeolites – crystalline and nanoporous aluminosilicates – the mordenite framework (IZA code: MOR) is one of the handful of synthetic zeolite structures used extensively in petrochemical industrial applications. Together with Faujasite (FAU) and ZSM-5 (MFI) zeolites, mordenite makes up the bulk of industrial zeolite use,^1^ owed to their high thermal stability, shape selectivity and manufacturing process excluding the use of expensive organic structure directing agents.^2^ Mordenite is used in the dewaxing process to upgrade paraffins *via* hydroisomerization as well as extensively in BTX processes such as benzene alkylation, toluene disproportionation and xylene isomerization.^2,3^

Mordenite zeolite was discovered as a mineral in Morden, Nova Scotia (Canada) by How in 1864 long before being synthesized hydrothermally in 1952.^4–6^ Mordenite is classified as a large-pore zeolite characterized by 12-rings that run along the *c*-axis, terminating at the [001] surface.^7^ Parallel to these 12-rings, there are highly compressed 8-rings with an aperture of 5.7 × 2.6 Å that are too small for molecules larger than 3.4 Å to enter. Orthogonally, along the *b*-axis there is another 8-ring with less distorted dimensions that intersects the compressed 8-ring channel and the 12-ring. It does not, however, create a two-dimensional system, as the 8-ring channels along the *b*-axis are staggered, making it impossible for large molecules to diffuse fully along the *b*-axis. Instead, the 8-ring channel along the *b*-axis is classified as a side-pocket that has been found to be highly selective for chemical transformations of small molecules such as dimethyl ether carbonylation and selective methane oxidation.^8–11^

A peculiarity of the mordenite zeolite is its existence in two variants, defined primarily by their port-size (*i.e.* the aperture of the pore system). This has been identified early on with the first mention of the phenomenon in the synthesis work by Sand.^5^ Small-port mordenite accepts less than 5 wt% of molecules larger than 4.2 Å (*e.g.* benzene) whereas large port mordenite achieves a higher uptake (>5 wt%/*ca.* 500 μmol g^−1^). Note that in both cases the crystal structure consists of 12-rings. Typical synthesis procedures for small-port mordenite involve high temperatures (275–300 °C), whereas the large-port mordenite can be crystallized at lower temperatures (<260 °C).^5^ Over the course of mordenite zeolite history, several tentative explanations for the origin of this feature have been proposed, such as localization of extra-framework cations, residual amorphous material in the pores, and stacking faults disrupting the continuity of the 12-ring channels.^5,12^ However, no conclusive evidence has been found to fully support one of these. It is noteworthy that dealumination leads to the opening of the framework transforming small-port into large-port mordenite.^13^ This points to the significance of Al placement within the framework.

**Fig. 1:**
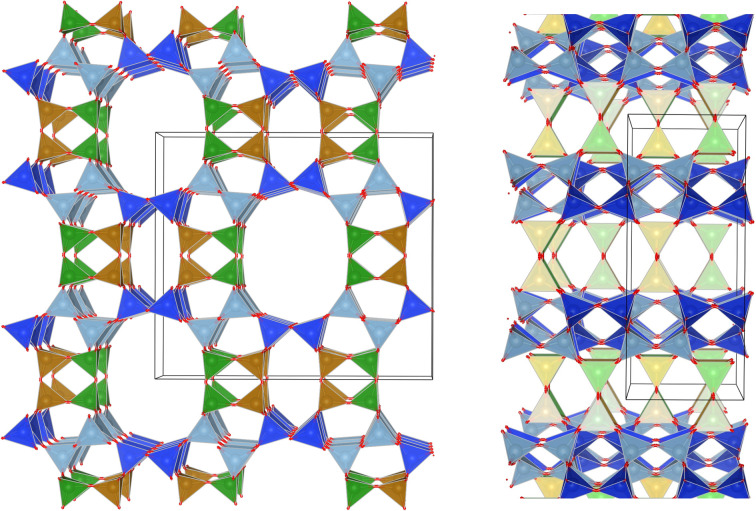
MOR framework illustrated by its corner sharing tetrahedral of SiO_4_ and AlO_4_. The T-sites are color-coded: 
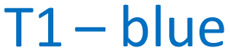
, 
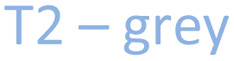
, 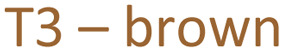, 
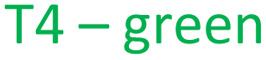
. (left) Shows the [001] surface while (right) shows the [010] surface.

Several studies have reported that among the 4 tetrahedral crystallographically non-equivalent sites, the T3 site (brown, Fig. 1) is preferentially occupied by Al.^14,15^ Simoncic & Armbruster found that in naturally occurring mordenite, Al in T3 has an occupancy of 0.41 while Al in T2 has an occupancy of only 0.03.^15^ This was associated with the high concentration of Ca^2+^ present in the material, which was found by structure refinement to be located within the center of the compressed 8-ring. The high charge localization had to be balanced by incorporating Al at the T3 position in the 8-ring.

This preferential localization of the Al and resulting negative framework charge determines the subsequently generated acid site centers and more general ion exchange site locations. From this, a profound impact on acid-catalyzed transformations follows; in carbonylation reactions it has been found that the confined space of the 8-ring makes the transition state, required for a highly selective carbonylation of MeOH and DME, feasible in the first place.^8,9^ Acid sites in the 12-ring, instead lead to coking. Similarly, the small molecule methane is proposed to be activated on Cu-oxo sites situated in the confined spaces of the 8-ring side-pocket.^10,11^ We hypothesize that Al siting can also explain the port size variation observed for mordenite.

Influencing Al placement in zeolites has been an ongoing effort over the last decade.^16–20^ This typically involves exploiting the charge density mismatch between large organic ammonium cations and small inorganic cations. This works primarily for high-silica zeolites (Si/Al > 10). A challenge arises for systems with Si/Al < 10 (*e.g.* mordenite). Influencing Al placement during zeolite synthesis, as opposed to post-synthetic modification,^21,22^ can only be achieved by varying the inorganic species present (*i.e.* Si source, Al source, mineralizer agent).^23,24^ Our previous contribution studied the impact of the Al source in the case of MOR.^25^ Small pH variations led to speciation of the Al location in the final material, which allowed us to deduce a structure–activity relationship for production of methanol from methane upon exchanging copper into the zeolite.^25^

Here we propose an alternative approach to influence Al distribution by stimulating synthesis of crystalline mordenite in the presence of cations of different size, namely sodium and potassium cations. With this we aim also to resolve the issue of port-size variation in this highly relevant catalyst system.

## 2. Results and discussion

### 2.1. Systematic investigation of cation nature on synthesis behavior

Our preliminary studies (ESI Note 1†) identified a feasible synthesis approach to obtain phase pure K-MOR using a sodium free silica source (Ludox AS-40, SiO_2_/Na_2_O > 470). Note, the crystallization temperature was 185 °C, far from the high temperature synthesis reported for small-port mordenite.^5^

**Fig. 2:**
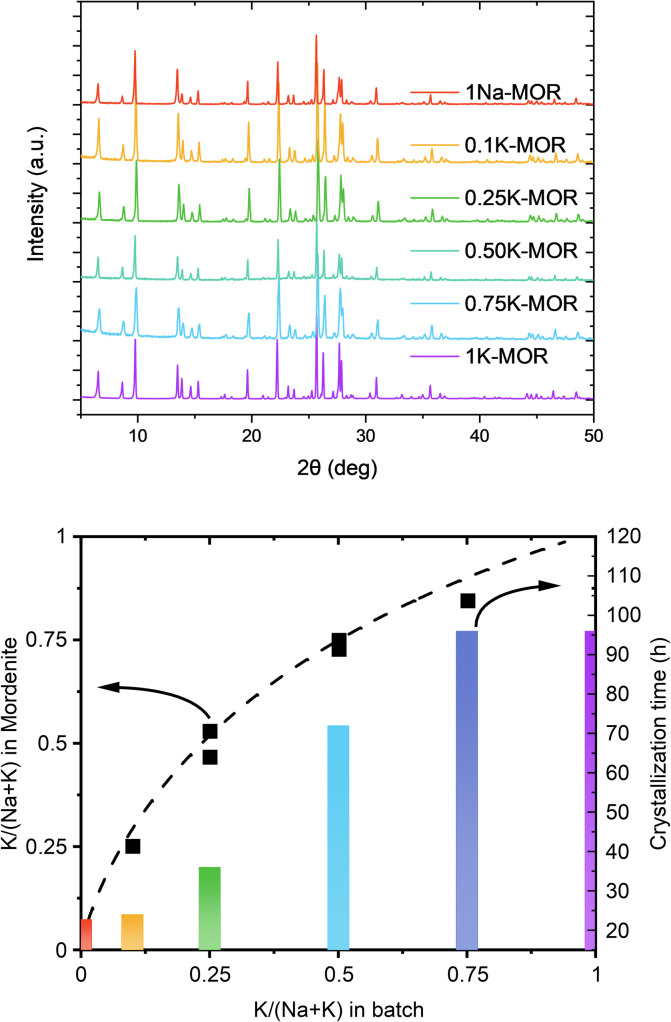
Top: The X-ray diffractograms measured for the fully crystalline samples. Bottom: A graph showcasing the K concentration between the gel and the dried mordenite zeolite as well as the corresponding times to achieve full crystallinity.

Next, we introduced NaOH and systematically varied the Na^+^/K^+^ ratio in the synthesis gel, while keeping the overall hydroxide concentration constant. In all cases we achieved phase purity, assessed by X-ray diffraction (Fig. 2, top), albeit it requires prolonged periods as the K^+^ content in the gel increases (Fig. 2, bottom). The final materials had comparable Si/Al ratios (*ca.* 6.7–7.3, Table S1†). Based on the amount of K^+^ incorporated in the crystalline material relative to that in the gel, there is a clear preference for K^+^, as shown by the convex deviation from the parity line (Fig. 2, bottom).

A closer look at the crystallization curves for the two extremes, reveals discrete differences with respect to induction times and crystallization rates (Fig. S3†). We find that sodium increases both the nucleation and crystallization rate. Whereas K-MOR has an induction period of *ca.* 15 h, preparing the same synthesis gel solely with NaOH already yields 30% crystallinity at the same time. During the induction period, the gel achieves supersaturation and the secondary building units present become thermodynamically equilibrated after which it becomes feasible to start growing crystallites, rather than the nuclei being dissolved by the highly alkaline conditions.^26,27^ The end of the induction period, signaled by the detection of X-ray diffracting crystals (*i.e.* domains > 10 nm),^28^ is followed by a period of crystal growth where dissolved aluminosilicate and silicate species condense with the growing crystals. Here the rate of crystal growth is mainly determined by the rate of depolymerization/hydrolysis of the Si source and condensation with the nuclei and crystallites present. With crystallite size being comparable across all samples (*vide infra*), the number of nuclei formed must be similar and the difference in crystallization curve must come from a slower crystal growth rate for KOH. The rate of depolymerization (and condensation/hydrolysis reactions needed for zeolite formation in general) is influenced by the concentration of hydroxide ions (pOH) and is thus directly linked to the pH, as shown by us previously.^25^ And yet, the K-MOR system crystallizes more slowly despite having a somewhat higher gel pH (Table S1†). This suggests that the nature of the cation has an outsize effect on zeolite formation. Naturally, the concentration of hydroxide ions would influence the rate of formation, however, in such concentrated gels the ionic strength of the base cannot be neglected. In fact, the viscosity of the gels was notedly different depending on the mineralizing agent, with the Na-MOR gel having a lower viscosity. This suggests NaOH to be a stronger mineralizing agent, achieving a faster depolymerization of the SiO_2_, releasing H_2_O and as such achieving lower viscosity. It is conceivable that the highly fluid Na-MOR gel allows for a rapid solid/liquid transport, thus also favoring crystallization. Furthermore, the aluminosilicate oligomers are in close contact with the charge balancing alkali cations and the interaction strength of these ions will also influence the oligomers formed. NMR studies by McCormick *et al.* have shown how larger cations can stabilize larger silicate oligomers.^29,30^ The affinity towards water cannot be neglected either, as this will determine how easily aluminosilicate species can condense with one another by displacing water molecules in the cation's hydration sphere.^31,32^ Breynaert *et al.* have recently shown how cation–oligomer interactions (*e.g.* Na^+^*vs.* Cs^+^) can influence crystallization behavior in high-silica ionic liquids (HSIL),^33,34^ while Wakihara *et al.* showcased this for Cs^+^ in the RHO framework.^35^ In addition, Okubo *et al.* have previously observed an elongated crystallization time for K-FER relative to Na-FER, which they attributed to a less facile stabilization of small primary building units (*e.g.* 4-rings) in the FER system.^36^ We believe a similar effect to influence the crystallization behavior in mordenite (*vide infra*).

### 2.2. Crystal morphology and refinement

**Fig. 3:**
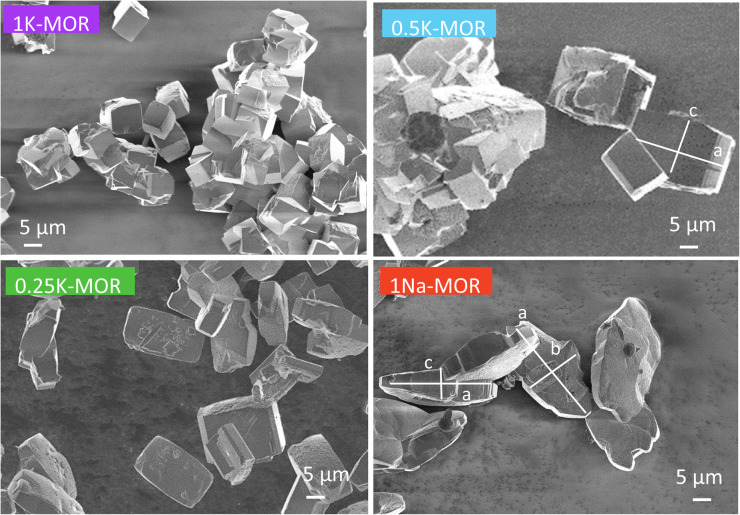
SEM images of a range of fully crystalline MOR zeolite with varying degrees of K incorporation.

Following the differences in crystallization behavior, it comes as no surprise that the morphology of the phase pure mordenite crystals also varies as seen from the SEM images shown in Fig. 3. 1Na-MOR crystallizes preferentially in prismatic shapes with an aspect ratio (*c*/*a*) of 0.28 (Fig. S4†).

**Fig. 4:**
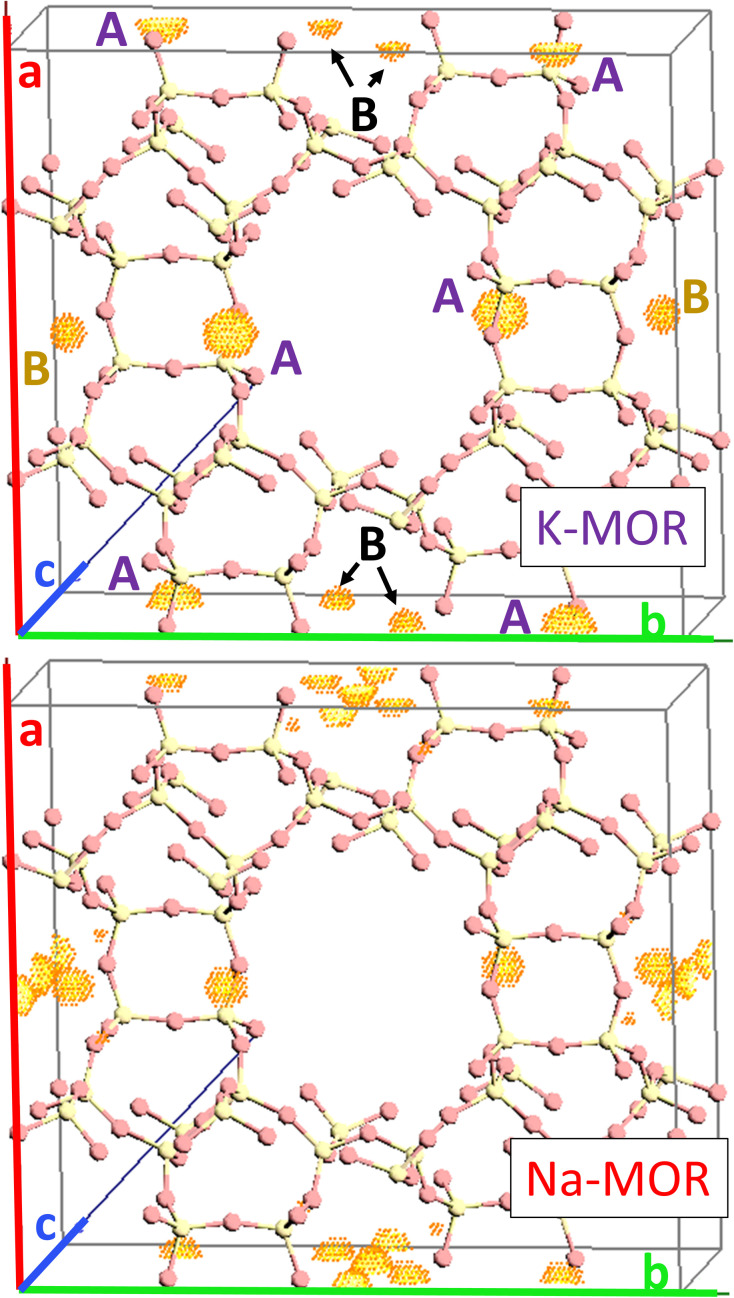
Difference Fourier map between structure factors calculated of a pure silica MOR empty framework and the dry alkali form of pure potassium sample (top) as well as the dry alkali form of pure sodium sample (bottom). The unique electron density clouds have been labeled as site A which is oriented towards 12-ring and site B inside the compressed 8-rings.

As the K-content in the gel increases the *a*-axis shrinks, increasing the aspect ratio to 0.49 for 0.5K-MOR and reaches unity as the morphology of the crystals becomes cubic for the 1K-MOR material. At the same time, the overall crystal size (volume assessed based on prismatic or cubic shape, Fig. S4†) remains comparable for all materials, despite a longer crystallization time for the K-enriched mordenite crystals. This suggests preferential facet growth in direction of the *c*-axis is taking place. We speculate this to be related to thermodynamically favored adsorption sites for soluble oligomers along the *c*-axis, stabilized by the K^+^ cations possibly due to their larger size. The structure directing role of cations has been postulated for other zeolites such as Na^+^ cations in FAU.^31,32,37^ This enrichment of potassium in the crystals occurs during synthesis and does not lead to a difference in Si/Al. For example, it is conceivable that the larger size of K^+^ cations will lead to less Al incorporated within the framework.^33^ With comparable Si/Al, this instead suggests a differentiation in where the Al is incorporated. Powder X-ray diffraction supports this hypothesis. The initial data across the samples already showed a clear trend in the structure factors variation for given reflections both in alkali-MOR and H-MOR (Fig. S5†). Thus, we decided to investigate this further on select samples, within a controlled environment of sealed borosilicate glass capillaries. We have repeatedly shown that it is possible to quantitatively determine the amount of coke or guest molecules inside the channels of the zeolite with Rietveld refinement of powder X-ray diffraction, by using dummy atoms as placeholders for residual electron density.^38–40^ Here we apply a similar approach for the alkali-MOR forms to achieve striking visual evidence of varying ion distribution, Fig. 4. As the cations – and by extension, the protons – are balancing the negative framework charge induced by framework Al this would strongly suggest a difference in the Al siting. Note this analysis is performed on flame sealed samples after water removal with refinement of hydrated samples resulting in a poorer fit (Fig. S6†). Dry K-MOR has two crystallographically unique alkali positions identical to the ones reported for rehydrated Ca-exchanged mordenite;^41^ site A, in the mouth of the side-pocket oriented towards the big 12-ring channel and site B, in the compressed 8-ring channel. In contrast, Na-MOR has three positions, of which two are in the compressed 8-ring channel and the last is very similar to site A of K-MOR.

While the Fourier maps suggest that site B is preferential in the Na-MOR, the residual electron density attributed to K^+^ ions is split more evenly between the 8-ring (site B) and 12-ring (site A). This might suggest that the steric constraint of a larger cation drives more of these charges to lie towards the 12-ring. Rietveld refinements now including Na^+^ and K^+^ in the models (see Fig. S6† to evaluate the quality of the fits) indicated that the distribution of the cations described as *site A/B ratio*, varies across the series accordingly; 1.01 for K-MOR, 0.72 for 0.5K-MOR and 0.39 for Na-MOR. Note, this trend follows the aspect ratio *c*/*a* ascertained by SEM, supporting the claim that larger K^+^ cations in site 1 favor crystal growth in *c* direction while site 2 favors the *a* direction. Indeed, this difference in crystal shape can also be observed when considering anisotropic crystallite shape parameters during Rietveld refinement. While they are typically limited to sizes of 100 nm,^42^ their inclusion for the more prismatic crystals of Na-MOR did improve the fit and does suggest a shrinking of the crystal shape in the *c* direction.

The presented powder diffraction data does not contain enough information to refine aluminum occupancies across T sites. However, assuming equivalence between alkali cations and Al allows us to measure a total amount of 5.4 potassium cations per unit cell, equal to a Si/Al ratio of 7.8. For the 0.5K-MOR and 1Na-MOR samples the Si/Al ratios are 5.9 and 6.2, respectively, agreeing sufficiently well with MP-AES results (Table S1†). Based on the two sites defined for K-MOR we used a rough estimation correlating these M^+^ sites with nearby T-sites (Fig. S7 and Table S2†). Interestingly, Fan *et al.* using ^23^Na MAS NMR claimed to observe 3 sodium sites: I, IV & VI and assigned them to T3, T4 and T1+2 respectively,^43^ however, we do not see any evidence of electron density around position VI (12-ring).

We can then use the occupancies of M^+^ in sites A and B to speculate about a different aluminum distribution, with total amount of M^+^ equaling Al. Site A is related to T2 & T4 while T3 & T1 are closer to site B. In reality the situation is more complicated because T1, although far away from site A, is also part of the 12-ring and oxygen bridging between T3 & T4 is arguably close to both M^+^ sites. As Al will occupy T-sites that are energetically most favored, our *site A/B ratio* obtained from refinement allows us to propose occupancies for T3 that go from 0.18 in the K-MOR to 0.33 in Na-MOR, while T4 for example is 0.20 in K-MOR and 0.14 in the Na-MOR. Note that while the T3 occupancy for Na-MOR is close to that reported in literature, we need to stress that this is based solely on electrostatic logic and only describes the Al distribution trend, rather than being an absolute numerical answer.

Lastly, there is a clear progression for the lattice parameters in both dry and hydrated states that suggests a major structural change when the big alkali cations are present (Fig. S8†).

### 2.3. The state of Al

We performed ^27^Al MAS NMR experiments on the two series endmembers to investigate the presence of extra-framework Al species. In the as-made materials only tetrahedral Al (55 ppm) is present (Fig. S9†) while the protonic form exhibits octahedral Al (0 ppm) as well. The amount of octahedral Al is 18% and found to be the same for both samples. The field strength of the instrument is unfortunately not high enough to resolve different T-sites either directly,^44^ or *via* MQMAS (not shown).^22^ The main distinguishing property between the materials is a peak shift, markedly more pronounced in the alkali-form. The tetrahedral peak of 1K-MOR is shifted by 0.5 ppm upfield and also has a narrower linewidth. The linewidth is influenced by the size of the quadrupolar coupling constant (QCC) with less distorted or more crystalline systems exhibiting smaller constants reducing the linewidth. The presence of spinning side bands (Fig. S9†) does suggest a more crystalline nature of 1K-MOR than 1Na-MOR, likely due to the longer crystallization time and cubic morphology. Additionally, a lower QCC would also result in a peak shift, however, downfield (to higher ppm).^45^ Instead, the peak is shifted upfield, which would suggest a difference in Al site distribution underlying the overall peak, supporting the findings from X-ray diffraction.

### 2.4. Sorption properties

**Fig. 5:**
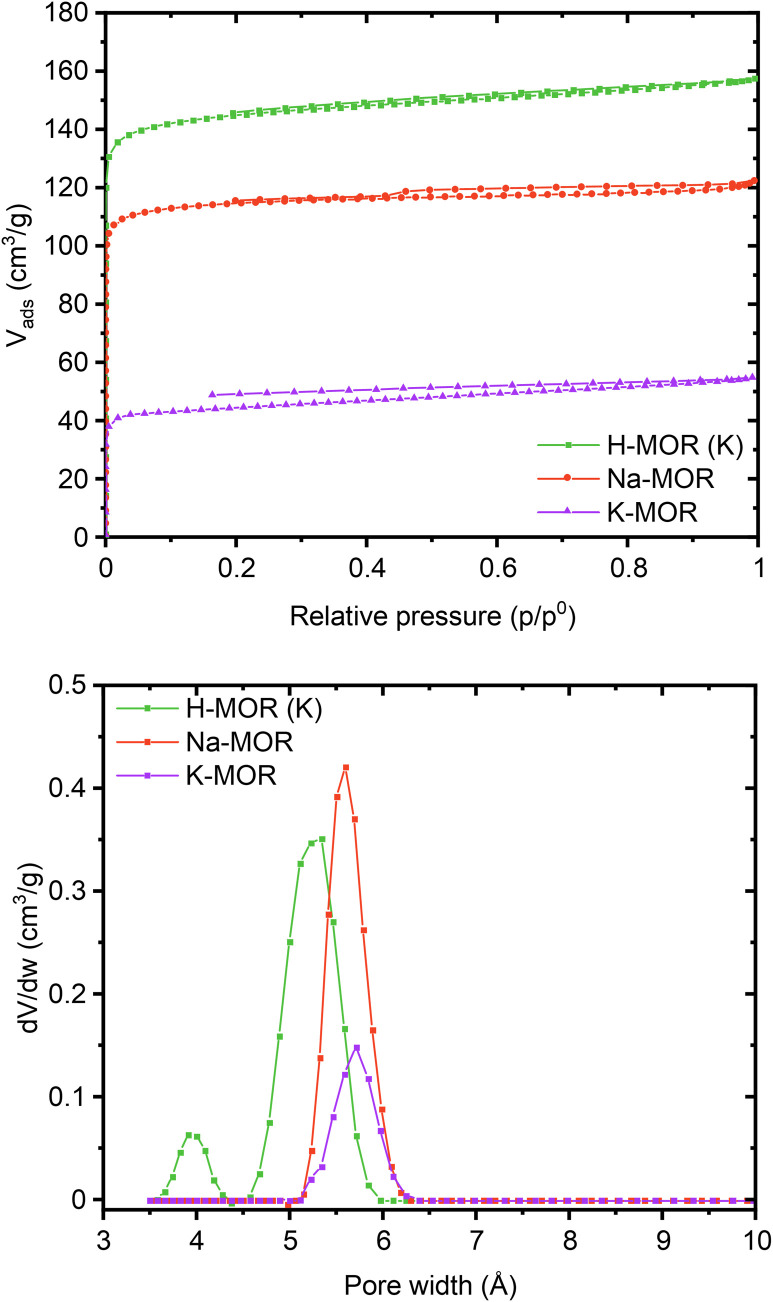
Ar physisorption isotherms (top) and pore size distributions (bottom) obtained at 87K for a select number of samples (Na-MOR, K-MOR, H-MOR (K)).

Encouraged by the clear morphological and cation siting differences, we then investigated how this impacts the intrinsic properties of the materials. The porosity of the as-made alkali-MOR was assessed with the help of N_2_-physisorption (Fig. S10 and Table S3†). The micropore volume and surface area increase with Na content. Due to the 8-ring in the [001] plane being distorted, the zeolite behaves akin to a 1-dimensional system when being probed with molecules as small as N_2_.^46^ Most interestingly, when measuring the porosity of the protonic zeolites, they all exhibit comparable surface areas and pore volumes (500–580 m^2^ g^−1^). Note, lattice parameters also showed a stark difference in the alkali form but more muted in the protonic form (Fig. S8†). Since all samples are fully crystalline mordenites, we conclude that the differing degrees of porosity in the alkali-form must relate to pore blockage due to the size of the cations. N_2_ possesses a quadrupolar moment and can thus weakly interact with cations present in the system.^47,48^ We thus corroborated our results by performing the sorption measurement for selected samples using liquid Ar instead (Fig. 5). The same trends are observed with this technique, K-MOR having the lowest and H-MOR the highest surface area. With the available high-resolution data obtained at very low pressures we were able to obtain a micropore size distribution (Fig. 5, bottom). Both alkali forms have pore widths of 5.6 Å, the Na-MOR sample having a larger porosity. The H-MOR sample, on the other hand, exhibits a bimodal distribution with 3.9 Å pores contributing a small amount of porosity. This is in line with the idea of the 8-ring side-pocket being accessible only *via* the 12-ring, blocked by the alkali cations in the case of the alkali form. Transforming the Na- into the K-form *via* ion exchange also leads to a lower porosity, further pointing towards cation induced pore blockage (Fig. S10,† bottom). The evidence obtained from the sorption properties is in agreement with the cation siting observed with X-ray diffraction and we next investigated whether this also leads to a variation on the port-size.

### 2.5. Towards probing the port size

**Fig. 6:**
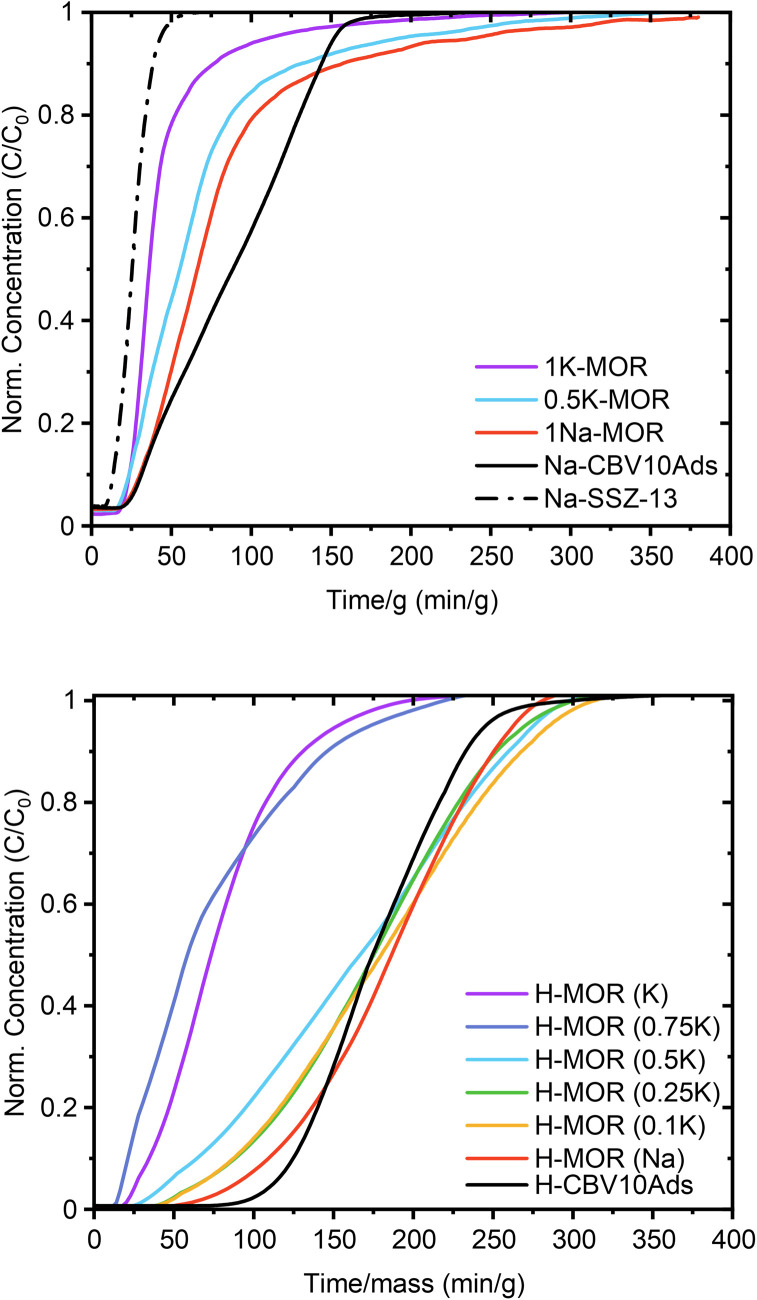
Toluene breakthrough curves for synthesized MOR in the alkali form (top) and the protonic form (bottom).

Sand who investigated mordenite synthesis in the 1960s first reported a variation in the port size. He described that the aperture of specific mordenite crystals adsorbed only small amounts of large molecules (kinetic diameter > 4.2 Å) whereas others achieved significant uptake (>500 μmol g^−1^).^5^ The materials were classified as small-port and large-port variants, respectively. Recently these terms have resurfaced with Knorpp *et al.* reporting that copper exchanged into large-port MOR is a superior selective methane oxidizer.^49^ Here we tested the port size by performing a toluene (kinetic diameter 5.85 Å) breakthrough experiment (Fig. 6).^50^ How quickly the toluene saturates the adsorbent bed is determined by the uptake capacity, which we can measure knowing the toluene vapor pressure. Thus, we find that the uptake is highest for the commercial MOR and 1Na-MOR (>500 μmol g^−1^) while MOR with more than 50% K^+^ in the gel behaves as a small port variant (264 μmol g^−1^). Note, these breakthrough curves refer to measurements carried out on the as-made alkali form, reported in Table 1. However, breakthrough curves for the protonic forms shown in Fig. 6 (bottom), exhibit the same trend with only slightly larger values (corresponding uptakes Table S4†).

**Table 1:** Quantification of the toluene breakthrough curves and TPD sequence for alkali-forms

Sample	Toluene (μmol g^−1^)	Toluene_TPD_ (μmol g^−1^)	Toluene_Si_
1K-MOR	264	232	7.8%
0.5K-MOR	452	317	11.0%
1Na-MOR	531	464	13.4%
Na-SSZ-13	219	28	n/a
NaCBV10Ads	522	371	15.4%

Over the years, several hypotheses have been put forward to explain the port-size variation, such as amorphous silica residue in the pores, defects in the crystal planes due to stacking faults – limiting passage, and extra-framework cations.^5,12,51^ We first noted differences in the pore accessibility when interrogating the materials with N_2_-physisorption, which is very sensitive to extra-framework species (see Fig. S10,† bottom). For the alkali-MOR, the larger K^+^ led to a low porosity material, suggesting that extra-framework cations can indeed influence the port size. Alternatively, one could argue that the K-MOR zeolites are prone to deposits of amorphous silica. However, we can exclude both the role of cations and the presence of amorphous silica as the H-forms of the prepared materials were highly porous in all cases when probed with N_2_. We see a similar behavior when investigating toluene breakthrough, comparing toluene adsorption on both alkali- and H-forms. In the latter case, more toluene is adsorbed (*e.g.* 530 μmol g^−1^*vs.* 700 μmol g^−1^ for 1Na-MOR) which matches the increase in porosity. However, this study is also proof that port-size cannot be ascertained by N_2_-physisorption alone, as the corresponding 1K-MOR sample experiences no increased toluene uptake when being transformed into the H-form.

We also exclude the role of defects. Stacking faults are associated with a higher concentration of silanol defects which can be identified by hydroxyl vibrations.^52,53^ Due to the large crystal size we applied a surface sensitive IR technique – DRIFTS – to compare the 1K-MOR and 1Na-MOR samples (Fig. S12†). However, we find no discernible differences between the two materials, other than perhaps that the H-MOR (Na) presents a slightly more intense band due to higher concentration of acid sites (3600 cm^−1^), and one could argue that the shoulder corresponding to external silanols (3735 cm^−1^) is marginally more pronounced.

Recently, Gao *et al.* have purported a simple technique to investigate framework Al in the channel intersections of ZSM-5.^54^ Performing a temperature programmed desorption with toluene on Na-exchanged ZSM-5, they were able to differentiate between toluene adsorbed on the siliceous pore wall and on framework Al (*i.e.* Brønsted acid sites).^54^ This differentiation can only be done due to the strong interaction between toluene and alkali cations and upon introducing an isothermal step at low temperature (100 °C). On the H-form, both adsorption sites are closer in strength resulting in peak overlap. Utilizing their approach, we observe similar features on the MOR system. The TPD profiles for the H-forms (Fig. S11†) present a low temperature shoulder, which we were able to resolve when using the alkali-MOR and a stepwise TPD profile. Clearly, a low temperature peak is observed for species held only weakly within the pores. Note, prior to the TPD, the sample is flushed for several hours in inert gas at 35 °C which removes some weakly bound toluene. This is evident when investigating the Na-SSZ-13 zeolite, which adsorbed 219 μmol g^−1^, but retained only a marginal amount of 28 μmol g^−1^ upon quantifying the desorbed toluene (Table 1), which follows from the large kinetic diameter of toluene (5.85 Å) relative to the 8-ring window of SSZ-13 (3.8 Å). Therefore, the amounts of desorbed toluene tend to be lower than those measured during the preceding breakthrough experiment.

Comparing the relative amount adsorbed on the siliceous pore wall to the toluene interacting with the framework Al in the 12-rings yields a crucial insight (Table 1). The small-port 1K-MOR has a much smaller fraction of toluene adsorbed on the siliceous pore wall (7.8%) compared to the large-port samples (13–15%). This is in agreement with the cation/Al siting results that imply a higher relative acid site concentration in the 12-ring for the K-MOR. There, a higher fraction of toluene will be interacting with cations, compared to the large-port, Na-MOR where most acid sites are located in the 8-ring.

Lastly, the shape of the breakthrough curve carries information relating to diffusional limitations.^55,56^ An ideal breakthrough curve has a clean front, indicating the absence of axial dispersion forces and intra-crystalline diffusion limitations such as that exhibited by SSZ-13 and 1K-MOR in Fig. 6. The large-port materials, on the other hand, present a more distended breakthrough curve, which implies the presence of diffusion limitations such as correlation effects between nearby adsorbed molecules.^56^ Note, that the Al–O bond is known to be slightly longer than the Si–O bond in zeolites (1.736 Å *vs.* 1.603 Å).^57^ This suggests that the higher concentration of Al in the side-pocket and 8-ring for the large-port mordenite likely expands this pore space. This allows for the pocketing of some toluene molecules interacting with cations and protons in these spaces, which increases their diffusion time and uptake capacity in the porous system.

### 2.6. Interrogating acid site properties

It is conceivable for the port-size to influence the confinement and reactivity of substrates in various acid-site catalyzed chemical transformations. We therefore investigated the acid site properties by adsorbing base molecules and subsequent TPD steps. Initially we decomposed the NH_4_-form of select samples to quantify the amount of NH_3_ released during the *in situ* formation of the protonic zeolite. We found this amount to match well with the total amount of Al from elemental analysis (Table S5†). Subsequent back-titration with NH_3_ vapors and performing a second TPD step resulted in the release of a lower amount of NH_3_, suggesting a reduced concentration of framework Al, owed to the formation of extra-framework Al during the *in situ* calcination of the NH_4_-form.^25^ The difference in NH_3_ released in the sequential TPDs is comparable across the range of samples suggesting no stark differences in Al framework stability.^25^ While we note no differences due to port-size for the NH_3_-TPD, we anticipated a larger base molecule to behave differently prompting us to perform propylamine TPD next. When adsorbed propylamine is heated above a specific temperature, Brønsted acid sites catalyze the Hoffman elimination leading to equimolar amounts of propene and NH_3_,^58^ both of which can be quantified to obtain the concentration of Brønsted acid sites. Propene is typically used for quantification, due to its narrow desorption peak shape, a result of negligible re-adsorption, unlike NH_3_. Table S6† summarizes the concentration of acid sites determined with the propene signal. For 1Na-MOR the agreement in acid site concentration between NH_3_-TPD and propylamine-TPD (propene quantification) is perfect, while it diverges significantly for 1K-MOR (and to a lesser extent 0.5K-MOR).

**Fig. 7:**
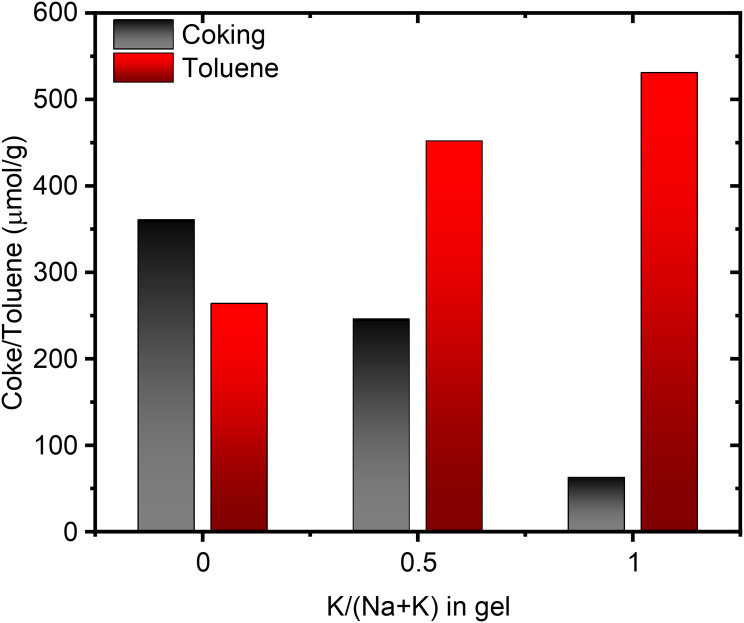
Correlation between the amount of coke produced during propylamine TPD and the amount of toluene adsorbed as a function of the potassium content in the synthesis gel.

However, the amount of NH_3_ released during the Hoffman elimination, is largely constant across all samples and in close agreement with the results from NH_3_-TPD (Tables S5 and S6†). This suggests the discrepancy between propene and NH_3_ for the K-enriched systems to originate from not all propene exiting the pore system. Dark coloration of the samples post-reaction, indicating coking, corroborates this. Interestingly, H-SSZ-13 (Si/Al 6), a small pore zeolite (8-ring windows) does not suffer from this carbon loss by coking, making our observation unique to the MOR framework, and more specifically unique to small-port mordenite. Indeed, when following the propylamine TPD with a subsequent temperature-programmed oxidation (TPO) step the coke can be oxidized to CO_2_. Quantification of the released CO_2_ allows us to account for a significant fraction of propene lost due to coking. A trend is clearly seen, evolved CO_2_ correlating with the amount of K in the gel during synthesis, *i.e.* the small port mordenites (Fig. 7). Meanwhile the small-pore SSZ-13 evolves only negligible amounts of CO_2_.

Most noteworthy, 1-dimensional zeolites (such as MOR, TON) favor the formation of linear oligomers over β-scission and isomerization reactions at 200–350 °C as investigated by Sarazen *et al.*^59,60^ They find that propene oligomerization rates increase dramatically with the pore constraints. These oligomers can then easily lead to the formation of coke by re-adsorption and protonation on nearby acid sites. We surmise the 8-ring windows in SSZ-13 – allowing access to a large cavity – to be insufficient to induce this oligomerization behavior. We aimed to reproduce the reported results, however, even at very low partial pressures of propene the deactivation was instantaneous (faster than equipment time resolution), irrespective of MOR tested (1K-MOR, 1Na-MOR), as reported in the ESI (Fig. S13†).

### 2.7. Port-size implication on alkane cracking

We concluded that the high acid site density in our MOR (Si/Al 6–7), their large crystal size, and the reactivity of propene prevent a detailed analysis of propene oligomerization rates. This demonstrates however the high activity of these MOR systems to oligomerize propene and form coke. Instead, we investigated the constrained environment with the help of the skeletal isomerization of isobutane as a probe reaction (Fig. S14†). In addition to the alkane being less reactive than the alkene, its steric nature (kinetic diameter = 5.28 Å) makes it sensitive to the pore environment. In fact, Gounder & Iglesia suggested that isobutane cracking is enhanced by acid sites located in the 8-ring due to partial confinement.^61^ Thus, we are able to assess the catalyst's active site confinement while retarding the deactivation as seen by its lifetime of several hours. Initially, isobutane is converted primarily to the linear isomer as well as undergoing disproportionation reaction to form C_3_ and C_5_ products. With gradual deactivation, hydrogen shift pathways become more prominent leading to the formation of isobutene. The in-house synthesized samples deactivate faster, which we attribute to their larger crystallite size. Crucially, however, the initial conversion is higher for large-port MOR than the small-port MOR. This is in line with the large-port MOR having more acid sites situated in the 8-ring, where partial confinement favors conversion. At the same time, we believe the expansion of the pore space due to enrichment in Al sites to become especially pronounced under dynamic conditions such as catalysis. This allows for not only pocketing more toluene, but likely also promotes the confinement of isobutane transition states. Note, in contrast to the work by Gounder and Iglesia working at differential conversions to elucidate reaction rates, our conversions tend be higher and the presence of C_5+_ products suggests the occurrence of bimolecular reactions, which should warrant a more thorough kinetic analysis in future contributions.

Additionally, the introduction of Al into specific locations likely also influences the flexibility and degree of distortion of pores. Researchers recently demonstrated how the adsorption of benzene can distort the 10-rings of ZSM-5 leading to a reversible 15% pore expansion.^62,63^ They related this to the flexibility of the Si–O–Si bond angles. Whether this facet of zeolite chemistry can have a profound influence on catalysis remains to be seen.^64^

## 3. Conclusions

Our results convincingly show that the cation nature present during the synthesis has a profound impact on the material outcome and its properties. The combination of Rietveld refinement, Ar-, and N_2_-physisorption data reasons for a higher concentration of K^+^ cations in the 12-ring, from which an Al distribution favoring the T4 sites in 12-ring follows. The consequence of this is the creation of a small-port MOR system shown by toluene breakthrough and stepwise TPD studies to behave akin to a one dimensional small-pore zeolite. As a result, alkenes such as propene undergo a more rapid oligomerization, explaining our propylamine TPD results (Fig. 7).

We can thus conclude that Al-siting lies behind the mysterious behavior of mordenite port-size variation, with more Al in the 12-ring leading to a small-port material. In fact, the works by Marcilly *et al.* highlight how acidic leaching of Al as a means of dealumination can lead to the transformation of small-port to large-port MOR. Once *ca.* 20% of the tetrahedral Al are extracted, the material's benzene uptake increases dramatically.^13^ This supports our claim that the population of framework Al in specific sites affects the port-size.

It is conceivable that the large spread in performance data obtained on Cu-MOR for the stoichiometric methane to methanol reaction can be partially explained by the facile ability of pores to accommodate the creation of active Cu-oxo species.^65–68^ This simple approach of varying the Al location in MOR, elucidated here, will prove promising when investigating these materials for their performance in small molecule activation.

## 4. Experimental methods

### 4.1. Zeolite synthesis

#### 4.1.1. Na to K-MOR

To synthesize MOR with different alkali cations we adapted a method reported by Chi & Sand.^69^ The gel composition is 2.6M_2_O : 1Al_2_O_3_ : 16SiO_2_ : 166H_2_O, where M stands for Na or K. In short, we first dissolve 7.67 g KOH (85% Sigma Aldrich) in 10 mL H_2_O followed by addition of 3.41 g Al(OH)_3_·H_2_O (50–57% Al_2_O_3_, Aldrich). The solution is stirred for 1 h before adding another 10 mL H_2_O and stirring for an additional hour. The solution should become clear. Finally, 12.6 mL H_2_O are added and the clear Al solution is slowly added to 52.5 g of Si sol (Ludox AS-40). The gel was then aged overnight under viscous stirring before being crystallized inside Teflon liners at 185 °C for 96 h. In the case of Na-MOR, the same procedure was followed, but using NaOH (Sigma Aldrich). The mixed Na, K systems maintained the overall molar amount of MOH, but varied the ratio between KOH/NaOH. After the sample was crystallized, the solid was separated from the supernatant by vacuum filtration and washed with hot water until the pH < 10. The samples are labeled based on the K-content in the gel (*e.g.* 0.75K-MOR is synthesized with 75 mol% KOH to 25 mol% NaOH for a total of 2.6M_2_O : 1Al_2_O_3_) To obtain the protonic form of the zeolite the sample was first ion exchanged at 70 °C in 1.0 M NH_4_NO_3_ solution for 12 h. This was performed a total of three times, before calcining the dried NH_4_-form at 500 °C for 6 h in static air.

#### 4.1.2. SSZ-13

The SSZ-13 zeolite was synthesized following the procedure reported by Prodinger *et al.*^16^ First 1.33 g NaOH (Sigma Aldrich) was dissolved in 110.4 mL H_2_O, followed by 2.16 g Al(OH)_3_·H_2_O (Aldrich) and trimethyladamantium hydroxide (25% in H_2_O) until a clear solution is obtained. To this clear solution, we slowly added 10 g of silica powder (Fumed SiO_2_, Aldrich) and then aged the gel under stirring for 24 h. The aged gel was allowed to crystallize at 160 °C for 96 h and then worked up by vacuum filtration until the solution pH < 10.

To obtain the protonic form of the zeolite the sample was first ion exchanged at 70 °C in 1.0 M NH_4_NO_3_ solution for 12 h. This was performed a total of three times, before calcining the dried NH_4_-form at 500 °C for 6 h in static air. In the case of the SSZ-13 zeolite the organic structure directing agent had to first be calcined, which was done by heating the as-made material to 550 °C in static air and holding it at this temperature for 6 h.

#### 4.1.3. Commercial MOR

The commercial MOR was received in its NH_4_ form (CBV10Ads, Zeolyst) and to obtain the protonic form, it was calcined following the same procedure as for the other zeolite samples.

### 4.2. Characterization

#### 4.2.1. X-ray diffraction

X-ray diffraction patterns for phase identification and the crystallization curves were collected on a Bruker D8 Discovery diffractometer using Cu-Kα radiation (*λ* = 1.5418 Å) experiments were run on a pressed powder sample holder in 2 theta range of 2–50° with a step size of 0.02° s^−1^ under ambient conditions. X-ray diffractograms for Rietveld refinements were recorded using a Bruker D8-A25 in transmission capillary geometry with Ge (111) Johanssen monochromator and Lynxeye detector with Cu K-alpha-1 radiation (*λ* = 1.5406). The samples were collected at 25 °C equilibrated with ambient moisture and after water removal at 300 °C for 3 hours on flame sealed capillaries.

#### 4.2.2. Solid state NMR

^27^Al MAS NMR measurements were performed on a Bruker Avance III spectrometer (11.74 T) located at SINTEF, Oslo (NO). Experiments were conducted in a 3.2 mm triple-resonance MAS probe at a spinning speed of 20 kHz. A single pulse sequence with a pulse length of 0.44 μs, corresponding to a 15° pulse angle and a recycle delay of 0.5 s, was used. A total of 10 000 scans were collected. The spectra were referenced to an aqueous Al(NO_3_)_3_ solution. The samples were hydrated over saturated Ca(NO_3_)_2_ solution for 48 h prior to the measurement.

#### 4.2.3. N_2_-physisorption

Isotherms were obtained using a BELSorp Maxi volumetric gas adsorption instrument (MicroTrac MRB) with at −196 °C. Prior to analysis the sample was evacuated at 300 °C for 10 h. The specific surface area was determined using the BET equation under pressure range relevant for microporous materials as defined by Rouquerol *et al.*^70^ Total pore volume was obtained at *p*/*p*_0_ 0.99. Micropore volume was calculated based on the t-plot method.

#### 4.2.4. Ar-physisorption

Isotherms were obtained on a quantachrome using liquid Ar at −186 °C. Pore size distributions were obtained with the SAEIUS software using the Ar 87K model for alkali and H-zeolites.^71^

#### 4.2.5. Scanning electron microscopy (SEM)

A Hitachi SU8230 microscope was used to obtain micrographs using an 1 kV acceleration voltage at 10 μA current.

#### 4.2.6. Diffuse reflectance infrared Fourier transformed spectroscopy (DRIFTS)

DRIFTS spectra were obtained on a Bruker Vertex 70 FT-IR spectrometer using a Harrick Praying Mantis cell. Prior to measurement, a reference sample (KBr) was placed in the sample holder and a background was collected. Then the reference sample was replaced by the material to be investigated and sample activation took place. The final spectra was collected at 380 °C after 1 h activation at 450 °C. The background from gaseous water was removed post collection.

#### 4.2.7. Temperature programmed desorption (TPD)

To probe the concentration of Brønsted acid sites, the acid-catalyzed Hoffman elimination of chemisorbed *n*-propylamine was studied in a homemade flow adsorption setup connected to a Pfeiffer Omnistar quadrupole mass spectrometer. First, the *ca.* 50 mg, pelletized protonic zeolite was activated for 1 h at 500 °C (10 °C min^−1^) in the flow of synthetic air (50 mL min^−1^). After cooling the sample to 170 °C the catalyst was exposed to saturated propylamine vapors in a N_2_ carrier gas (50 mL min^−1^) for 1 h. Physisorbed propylamine was then desorbed at the same temperature over the course of 4 h (66 mL min^−1^ N_2_) followed by heating to 650 °C (20 °C min^−1^) which decomposes the *n*-propylamine into propene and NH_3_. For select samples the TPD step was followed by a TPO protocol which involved cooling to room temperature and subsequently heating to 650 °C in a flow of synthetic air (66 mL min^−1^). To quantify the amount of propene, NH_3_, and CO_2_ released a calibrant gas was injected into the system at the end of the experiment.

NH_3_ TPD was performed on the NH_4_-Form of select samples to quantify the total amount of NH_3_ released during the *in situ* generation of the H-Form. The sample was heated to 650 °C (10 °C min^−1^, 66 mL min^−1^ N_2_) and held at this temperature for 1 h before cooling to 200 °C. Upon reaching this temperature the now protonic zeolite was exposed to 0.25% NH_3_ in N_2_ (40 mL min^−1^) for 4 h, followed by flushing in inert for an additional 4 h. Finally, the sample was heated in inert (66 mL min^−1^) to 650 °C (10 °C min^−1^) to release any adsorbed NH_3_. Quantification was achieved with calibrant gas.

#### 4.2.8. Toluene breakthrough

In a typical experiment we used 30–40 mg of an alkali or protonic zeolite sieved into a narrow fraction (125–160 mm) suspended on a quartz frit in a homemade flow reactor connected to an Omnistar quadrupole mass spectrometer. Prior to exposing the sample to toluene vapors it was activated by heating to 500 °C in synthetic air. The sample was subsequently cooled to 35 °C. Toluene vapors were carried through the sample using N_2_ as carrier gas (15 mL min^−1^) with Toluene being cooled to 0 °C in an ice bath. We used the Antoine equation to determine the vapor pressure of toluene at 0 °C. Breakthrough measurements were normalized by mass and we followed *m*/*z* 92 for toluene. Following the breakthrough measurement, select samples underwent a stepwise temperature programmed desorption step to determine the desorption temperature and amount. For this the toluene saturated sample was first flushed for one hour with inert gas to remove weakly bound toluene (25 mL min^−1^, N_2_). Subsequently the temperature was increased to 100 °C (10 °C min^−1^) and held at this temperature for 30 min before heating to 500 °C (10 °C min^−1^).

#### 4.2.9. Microwave plasma atomic emission spectroscopy (MP-AES)

The elemental composition of the samples was determined with the help of Agilent 4100 MP-AES instrument. Initially, samples needed to be solubilized by dissolving *ca.* 20 mg material in 1 mL 15% HF for one hour in a Teflon liner. Complete dissolution was verified by the absence of solid particles when shining a light through the thin Teflon wall. Excess fluoride anions were bound by the addition of 5 wt% H_3_BO_3_ and the solution was diluted to 50 mL with distilled water. Quantification of the Si, Al, Na and Cu contents were achieved using external calibration curves using commercial elemental standards.

### 4.3. Catalytic testing

The catalytic activity of the MOR samples for propene oligomerization and isobutane cracking were evaluated in a Microactivity Effi high pressure unit (PID Eng & Tech). The unit was provided with a packed bed stainless steel reactor of 6 mm inner diameter. The reactor was heated by a cylindrical oven and the temperature was measured by a type K thermocouple in the middle of the catalysts bed (max 3 cm length). To ensure the inert character of the steel setup the reactor tube was previously Sillcolloy coated (SilcoTek) and a quartz sleeve was used for the thermocouple. The reaction products were analyzed online in a Scion 456 Gas Chromatograph (GC) equipped with 1 TCD, 2 FID detectors and six columns (MolSieve 13X, HayeSep Q, HayeSep N, Rt-Stabilwax, Rt-Alumina/MAPD, and Rtx-1). In a typical run, 200 mg of the catalyst was loaded into the reactor supported in an inert bed of glass wool. Prior to the reaction, the catalysts were pretreated at 550 °C in air using a heating ramp of 10 °C min^−1^. Then, reactant flows were stabilized in a bypass mode (bypassing the catalytic bed) until a constant signal was observed in the GC. These bypass values were used to compute the carbon balance and estimate the coke yields.

#### 4.3.1. Propene oligomerization

Propene oligomerization reactions were carried out at 230 °C using different reaction conditions. Reactions at 5 and 2 bar were tested using different partial pressures of propene in N_2_ (0.05, 0.3 and 0.6 bar). High Gas Hourly Space Velocity (GHSV) values were aimed, ranging from 17 000 to 60 000 cm^3^ g^−1^ h^−1^.

#### 4.3.2. Isobutane cracking

Isobutane cracking was performed at 450 °C and atmospheric pressure. The experiments were carried out at the low reactant partial pressure of 0.15 bar diluted in N_2_ and aiming at a GHSV value of 30 000 cm^3^ g^−1^ h^−1^.

## Conflicts of interest

There are no conflicts to declare.

## Supplementary Material

TA-011-D3TA03444E-s001
