# Supplementary material for: Cation-induced speciation of port-size during mordenite zeolite synthesis
Source: J Mater Chem A Mater. 2023 Sep 28;11(40):21884–94. doi: 10.1039/d3ta03444e (PMC10581370; doi:10.1039/d3ta03444e)
Supplement: TA-011-D3TA03444E-s001 [file TA-011-D3TA03444E-s001.pdf]

Electronic Supplementary Material (ESI) for Journal of Materials Chemistry A.
This journal is © The Royal Society of Chemistry 2023

Supporting Information

Cation-Induced Speciation of Port-Size in Mordenite Zeolite Synthesis

Sebastian Prodinger*[1], Izar Capel Berdiell[1], Tomas Cordero-Lanzac[1] , Odd Reidar Bygdnes[1], Bjørn Gading Solemsli[1], Karoline Kvande[1], Bjørnar Arstad[2] Pablo Beato[3], Unni Olsbye[1], & Stian Svelle[1]

1) Center for Materials Science and Nanotechnology (SMN), Department of Chemistry, University of Oslo, 1033 Blindern, 0315 Oslo, Norway

2) SINTEF Industry, Forskingsveien 1, 0373 Oslo, Norway

3) Haldor Topsøe A/S, Haldor Topsøes Allé 1, 2800 Kongens Lyngby, Denmark

KEYWORDS. Cations, Zeolite formation, Mordenite, port-size

Table of Content

Supporting Note 1. Preliminary synthesis studies 3

Figure S1. 3

Figure S2 3

Crystallization behavior 4

Figure S3 4

Table S1 4

Crystal Morphology 5

Figure S4 5

X-ray diffraction 6

Figure S5 6

Figure S6 6

Figure S7 7

Table S2. 8

Figure S8. 9

Solid State NMR 10

Figure S9 10

N_2-physisorption 11

Figure S10. 11
Table S3 11
Toluene Breakthrough 12
Table S4 12
Toluene TPD 12
Figure S11 12
DRIFTS 13
Figure S12 13
Acid site characterization 14
Table S5 14
Table S6. 14
Investigation of constrained environment via catalytic test reactions 15
Propene oligomerization 15
Figure S13 15
Isobutane cracking 15
Figure S14. 15

Supporting Note 1. Preliminary synthesis studies

We previously synthesized MOR zeolites with the help of precipitated silica (Hi-Sil 233) and various Al-sources and NaOH as mineralizer. To obtain a potassium pure K-MOR, however, we need to perform the synthesis with as little Na impurities as possible. Consequently we used $Al(OH)_3xH_2O$ as the Al source and initially investigated our previously reported synthesis method, solely substituting KOH for NaOH. However, even after extended periods of time no crystallinity was observed requiring us to attempt the synthesis using a method reported by Chi and Sand.[1] The main difference with this method is the elevated crystallization temperature of 185 °C compared to 170 °C which is expected to significantly increase crystallization rate. As the Na content of the Hi-Sil 233 is too high (SiO_2/Na_2O >75) we were prompted to try the synthesis of 1K-MOR with Hi-Sil 915 (SiO_2/Na_2O >270) as well as Ludox AS-40 (SiO_2/Na_2O >470). We also investigated the effect of aging which typically influences particle size.[2] These preliminary experiments concluded that aging is a crucial step ensuring phase-purity. The SEM images show pristine cubic crystals for the aged Ludox AS-40 whereas in the absence of aging, the crystals are decorated with small platelike agglomerates (Figure S1). Comparing the crystallization curves for the two Si sources highlights the identical induction periods followed by diverging crystallization rates, reflecting the difference in reactivity of the Si source (Figure S2). It is likely that the slower crystallization rate results in the formation of impurities observed with both XRD and SEM (Figure S1). Therefore, we opted to use Ludox AS-40 as the Si source.

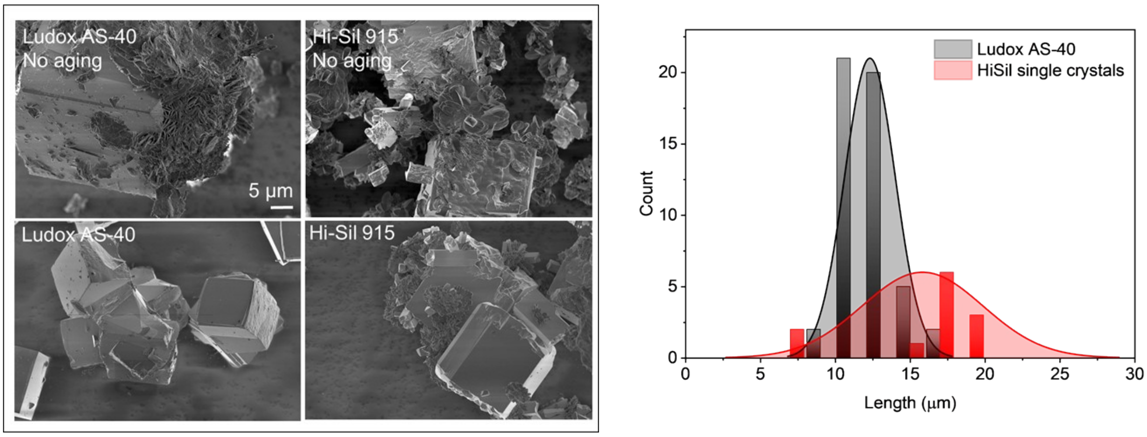

Figure S1. (left) Scanning electron micrographs of the fully crystalline (96+ h) K-MOR synthesized with two different silica sources in the presence and absence of aging (overnight). Only in the case of the aged gel, using Ludox AS-40 did we obtain cubic crystallites. (right) The particle size distribution suggests smaller and more uniform crystallites for Ludox AS-40 compared to using Hi-Sil 915 as Si source.

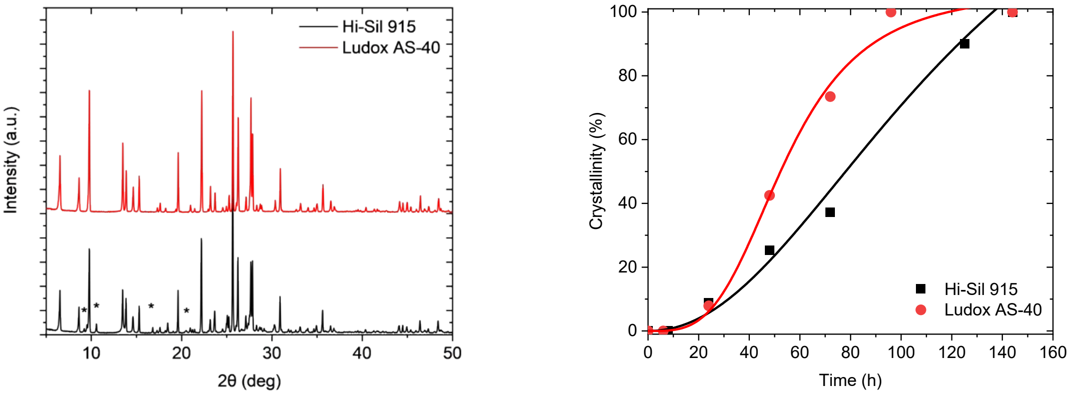

Figure S2. X-ray diffractograms of MOR crystallized with different Si sources (top). In case of using Hi-Sil 915, there are some phase impurities present. (right) Crystallization curve for K-MOR synthesized with the two different Si sources.

Crystallization behavior

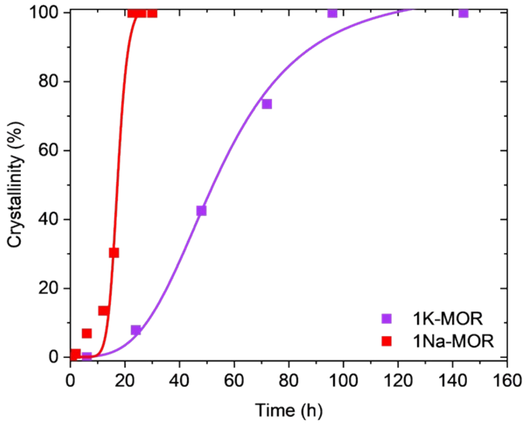

Figure S3. Crystallization curve for the K-MOR and Na-MOR systems with comparable gel composition and aging duration, solely differing on mineralizer agent nature. The data points are obtained by removing autoclaves at selected times and quenching the crystallization process. Crystallinity is assessed by comparing the intensity of X-ray diffraction peaks to the intensity of the broad peak related to amorphous silica (2θ =22°).

Table S1. Physicochemical characterization data for the synthesized MOR with varying amounts of Na and K in the gel. The solid yield is calculated with respect to the amount of SiO_2 utilized. Si/Al and Al content were determined from MP-AES and error margins are reported from performing multiple analyses across different batches. The average error margin for M^+/Al content is ca. 5%.

Sample	pH $_{Gel}$	Crystallization time (h)	Solid yield (%)	Si/Al $_{MP-AES}$	Al (µmol/g)	M^+/Al $_{MP-AES}$ K/Na
1K-MOR	14.3	96	77	7.0 ± 0.73	2089 ± 185	0.88
0.75K-MOR	n.d.	96	81	6.8 ± 0.43	2143 ± 120	0.82/0.15
0.5K-MOR	14.2	72	75	6.7 ± 0.23	2164 ± 66	0.69/0.23
0.25K-MOR	n.d.	36	71	7.3 ± 0.50	1999 ± 100	0.44/0.50
0.10K-MOR	n.d.	24	85	7.0 ± 0.45	2077 ± 93	0.24/0.71
1Na-MOR	14.0	22.75	98	7.3 ± 0.47	2023 ± 116	0.89

Crystal Morphology

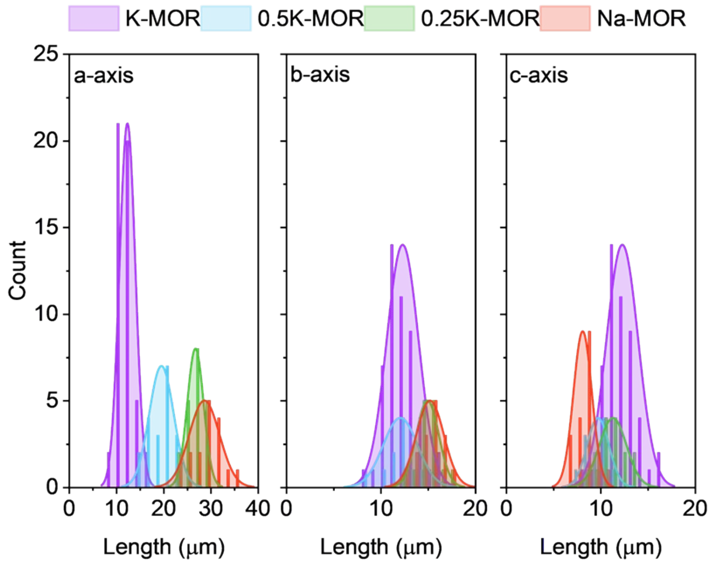

Sample	Crystal volume (μm³)	Aspect ratio (c/a)
1K-MOR	1867 ± 439	1
0.5K-MOR	2124 ± 450	0.49 ± 0.08
0.25K -MOR	3754 ± 590	0.42 ± 0.06
1Na-MOR	2210 ± 410	0.28 ± 0.04

Figure S4. Particle size distributions for select mordenite samples and the derived crystal parameters such as crystal volume and aspect ratio. For representative SEM images see main text Figure 3.

X-ray diffraction

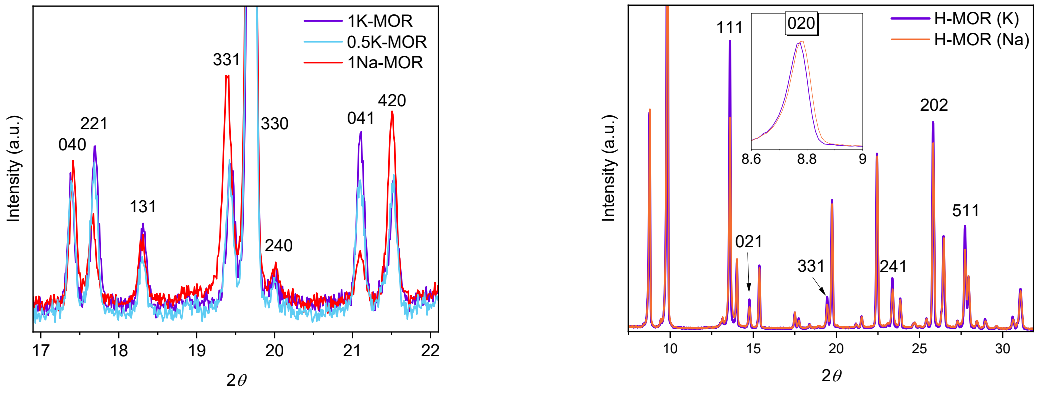

Figure S5. Unrefined, capillary XRD patterns of select M⁺-MOR samples (left). Unrefined XRD patterns of H-MOR of series end-members where the reflections showing intensities differences are highlighted (right).

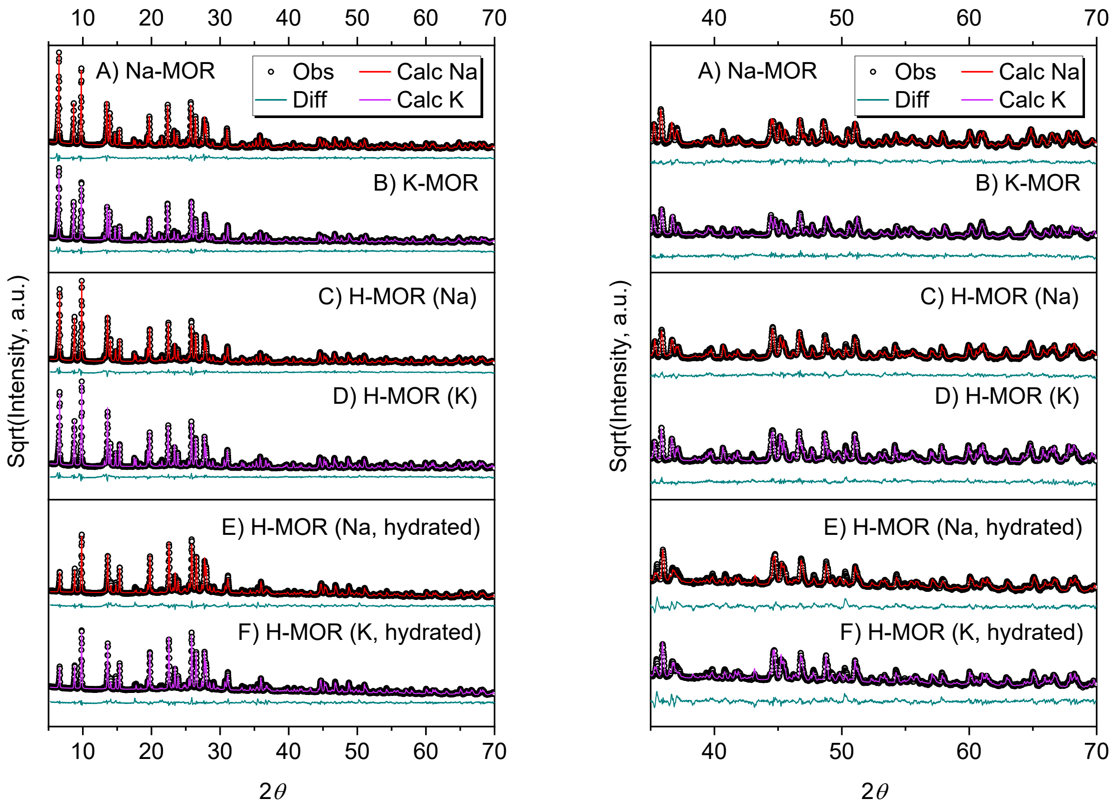

Figure S6. Rietveld refinements of the two end-members of the sample series in the three relevant states; M⁺-MOR (top), H-MOR (middle) and H-MOR hydrated (bottom). Red lines are the calculated model, the black circles represent the experiment and the green is error line, both panels show the same refinement but the right highlights the high angle fitting.

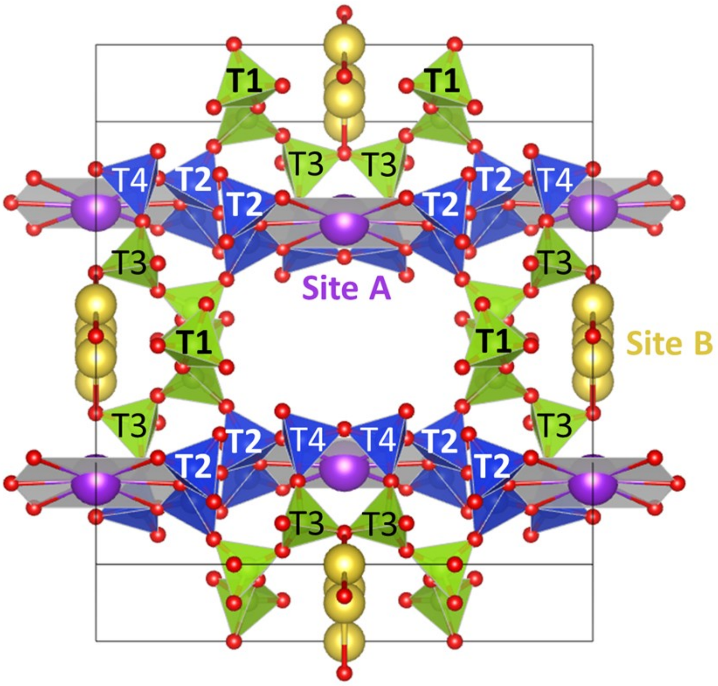

Figure S7. Description of M^+ sites A and B with the pairs of T atoms near each of them; Site A = T2 & T4, Site B = T1 & T3.

Based on the proximity estimation:

Equation 1: Site A: T2*16 + T4*8

Equation 2: Site B: T1*16 + T3*8

Mean site distribution T1*18, T2*10, T3*43, T4*29 would represent a site A/B ratio of 0.62 with 5.44, 6.96 & 6.64 total Al respectively the occupancies should be:

%Al (T1) = 0.0176*x = 0.0957 0.122 0.117

%Al (T2) = 0.0098*x = 0.0533 0.068 0.065

%Al (T3) = 0.0420*x = 0.228 0.292 0.279

%Al (T4) = 0.0283*x = 0.154 0.197 0.188

If we assume the *site A/B ratio* proportionality across the series, presumed Al occupancies can be calculated by a simple system of equations, given that T1/T3 ratios (0.419) and T2/T4 (0.345) remain constant within the relative occupancies. Values obtained are described in the table and forced in the final refinement models with negligible effect in the fittings:

Table S2. Overview of the Rietveld refinement parameters from capillary XRD measurements.

	Form	R_{wp}	*a* (Å)	*b* (Å)	*c* (Å)	cell vol (Å³)	Anis. Cryst. shape (b_{11} b_{22} b_{33})	Formula	M⁺ Site A	M⁺ Site B	Site A/B ratio	Dummy water	Presumed Al occupancies	Total Al nearby sites & A/B ratio
1K-MOR	**Alkali**	**10.4**	**18.0358(5)**	**20.3746(5)**	**7.46260(15)**	**2742.31(11)**	0.00000<br>0.00092<br>0.07609	$Si_{42.56}Al_{5.44}O_{96}$<br>(Si/Al: 7.8)	2.73	2.7	1.01	0	T1 = 0.077<br>T2 = 0.070<br>T3 = 0.184<br>T4 = 0.202	Site A = 2.74<br>Site B = 2.7<br>Ratio = 1.01
	M⁺(water)	13.1	18.1065(6)	20.4717(6)	7.50112(19)	2780.45(14)			3.0	2.18	1.37	23.2		
	H	10.5	18.1649(4)	20.3926(4)	7.49553(13)	2776.57(10)			-	-	-	0		
	H (water)	11.6	18.1778(6)	20.2993(5)	7.48064(18)	2760.33(12)			-	-	-	48.5		
0.5K-MOR	**Alkali**	**11.1**	**18.0502(5)**	**20.3745(4)**	**7.47141(16)**	**2747.71(11)**	0.00000<br>0.00000<br>0.18162	$Si_{41.04}Al_{6.96}O_{96}$<br>(Si/Al: 5.9)	K: 2.12<br>Na: 0.8	K: 1.04<br>Na: 3.0	0.72	0	T1 = 0.115<br>T2 = 0.074<br>T3 = 0.275<br>T4 = 0.216	Site A = 2.91<br>Site B = 4.05<br>Ratio = 0.72
	M⁺(water)	12.7	18.0964(6)	20.4456(6)	7.4971(2)	2773.85(14)			K: 2.28<br>Na: 0	K: 1.04<br>Na: 4.44	0.42	18.2		
1Na-MOR	**Alkali**	**10.9**	**18.0689(5)**	**20.3498(5)**	**7.49318(18)**	**2755.23(12)**	0.00039<br>0.00186<br>1.14957	$Si_{41.36}Al_{6.64}O_{96}$<br>(Si/Al: 6.2)	1.88	4.76	0.39	0	T1 = 0.136<br>T2 = 0.048<br>T3 = 0.325<br>T4 = 0.138	Site A = 1.87<br>Site B = 4.77<br>Ratio = 0.39
	M⁺(water)	12.5	18.0835(6)	20.4130(5)	7.5016(2)	2769.13(14)			2.01	5.67	0.35	17.2		
	H	11.5	18.1651(5)	20.3785(5)	7.4934(2)	2773.87(13)			-	-	-	0		
	H (water)	12.3	18.1736(7)	20.2830(6)	7.4792(2)	2756.93(15)			-	-	-	47.4		

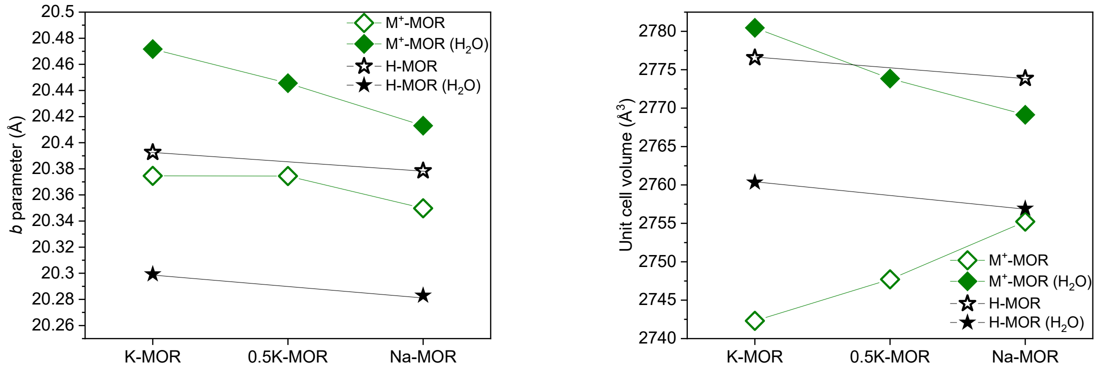

Figure S8. (Left) Evolution of *b* lattice parameter, (right) evolution of unit cell volume, across the samples, form and hydration.

The structural parameter differences became less significant for the protonic forms (Table S2 & Figure S8). The most significant difference between dry H-MOR samples of both extremes is *b* lattice parameter, which suggest that something must be different between their empty frameworks (See 020 reflection shift in Figure S5). However we are talking about a minimal difference in the unit cell volume; 2777 vs 2774 Å^3 for K- and Na-MOR, respectively. It has been suggested that the unit cell volume can be correlated to the Si/Al ratio.[3] However, we see quite a large variation within our measured samples of similar Si/Al ratios and it should be emphasized that water has an enormous effect so care must be taken when reporting and comparing these values.

Solid State NMR

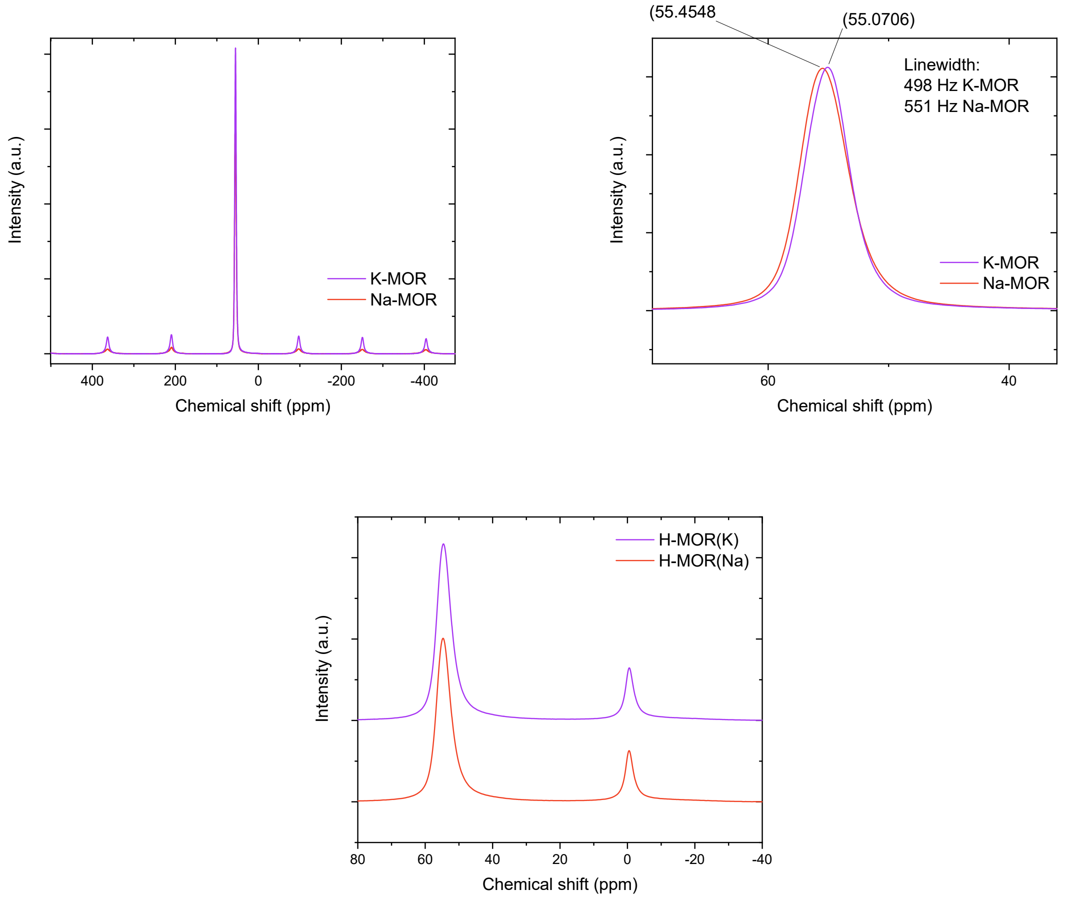

Figure S9. ^{27}Al MAS NMR spectra of the as-made (top left) and H-forms (bottom). Top right focuses on the peak shift observed for the as-made materials.

N_2-physisorption

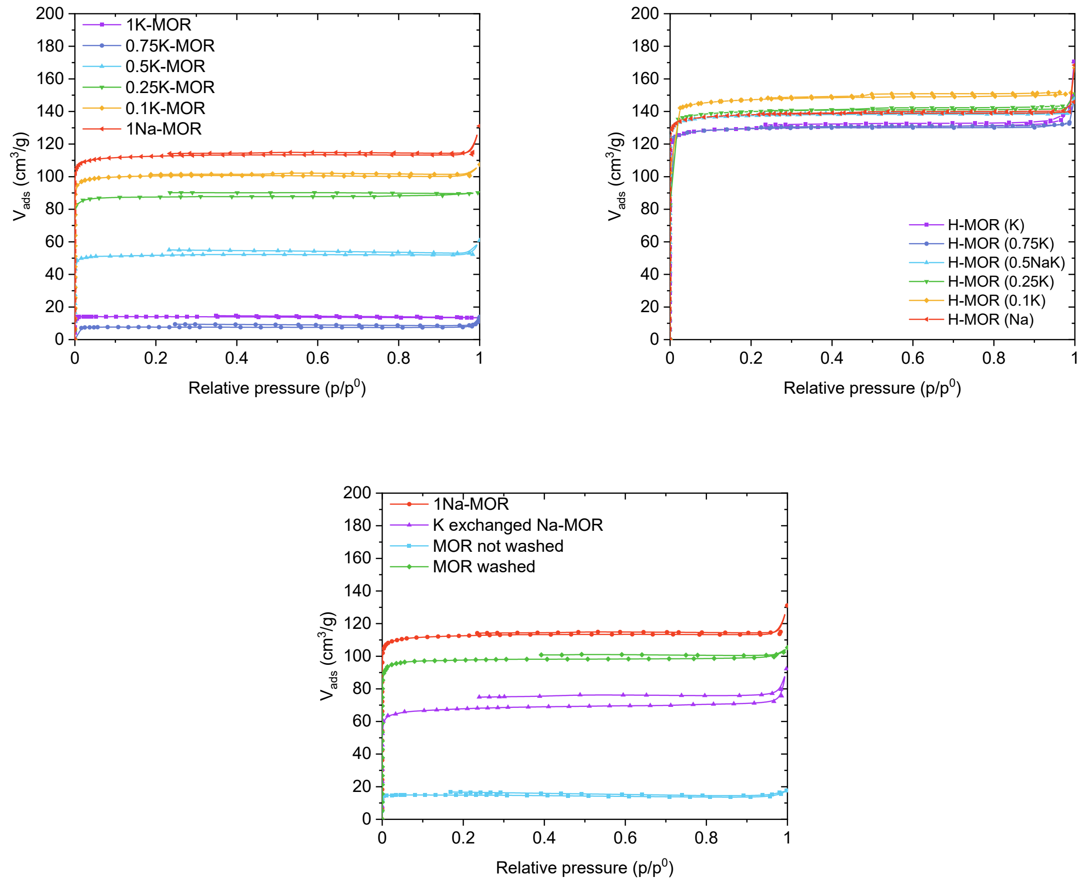

Figure S10. N_2 physisorption isotherms of the alkali MOR zeolite (left) and their protonic counterparts (right). It is clear that the presence and type of cations has significant impact on the measureable surface area. Bottom: isotherms for the Na-MOR and a K-exchanged Na-MOR sample. The larger size of the K^+ reduces the surface area. Also shown are two isotherms for as-synthesized MOR (different synthesis procedure) without washing and after washing with hot water. Elemental analysis of the first wash solution showed a high Si/Al ratio (650) whereas subsequent solutions had a lower Si content (Si/Al 39). At the same time the Si/Al ratio of the washed zeolite decreased from 6.6 to 6.5 suggesting amorphous silica deposits.

Table S3. Overview of the data gathered from N_2-physisorption on the as-made alkali zeolite and the subsequently generated H-form. The H-Form of all samples behave nearly identical.

	Alkali Form			H-Form		
**Sample**	**Surface area (m^2/g)**	**Micropore volume (cm^3/g)**	**Total pore volume (cm^3/g)**	**Surface area (m^2/g)**	**Micropore volume (cm^3/g**	**Total pore volume (cm^3/g)**
**1K-MOR**	60	0.02	0.02	565	0.21	0.22
**0.75K-MOR**	38	0.01	0.02	510	0.20	0.21
**0.5K-MOR**	215	0.08	0.08	570	0.21	0.22
**0.25K-MOR**	366	0.13	0.14	555	0.22	0.23
**0.10K-MOR**	417	0.15	0.16	580	0.23	0.23
**1Na-MOR**	465	0.17	0.19	573	0.21	0.23
**K-ex-1Na-MOR**	274	0.10	0.13	n.a.	n.a.	n.a.

Toluene Breakthrough

Table S4. Toluene uptake values obtained on the breakthrough curves of protonic MOR. Uncertainty ± 5%

Sample	$Toluene_{H\text{-}Form}$ (µmol/g)
**H-MOR (K)**	270
**H-MOR (0.75K)**	245
**H-MOR (0.5K)**	656
**H -MOR (0.25K)**	657
**H-MOR (0.10K)**	715
**H-MOR (Na)**	706
**H-CBV10-Ads**	692

Toluene TPD

On select samples we investigated the uptake behavior as well as desorption behavior as a function of the temperature. The alkali exchanged forms of the listed mordenite samples were exposed to toluene vapors (see main text) followed by a stepwise TPD sequence with an isothermal step at 100 °C. When performing this procedure on the protonic forms, the peaks overlap as seen in Figure S10.

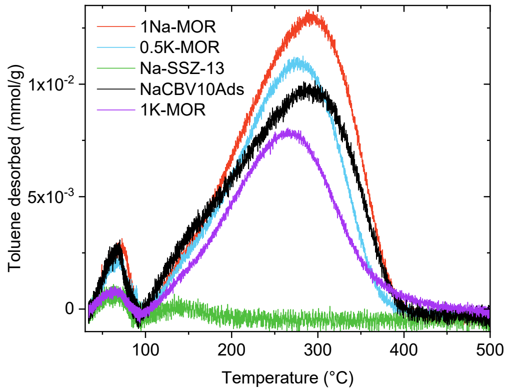

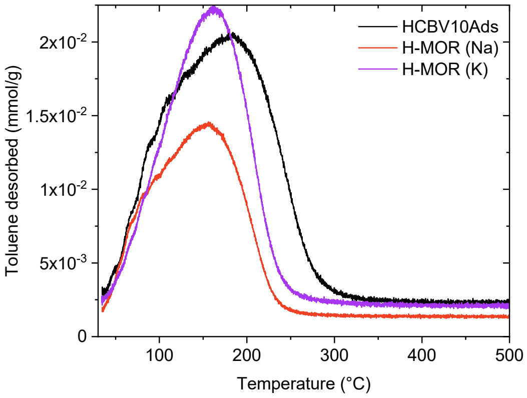

Figure S11. Toluene stepwise TPD performed on the protonic forms of various mordenites. In contrast to the alkali-form, the peaks assigned to siliceous pore wall and acid site overlap here.

DRIFTS

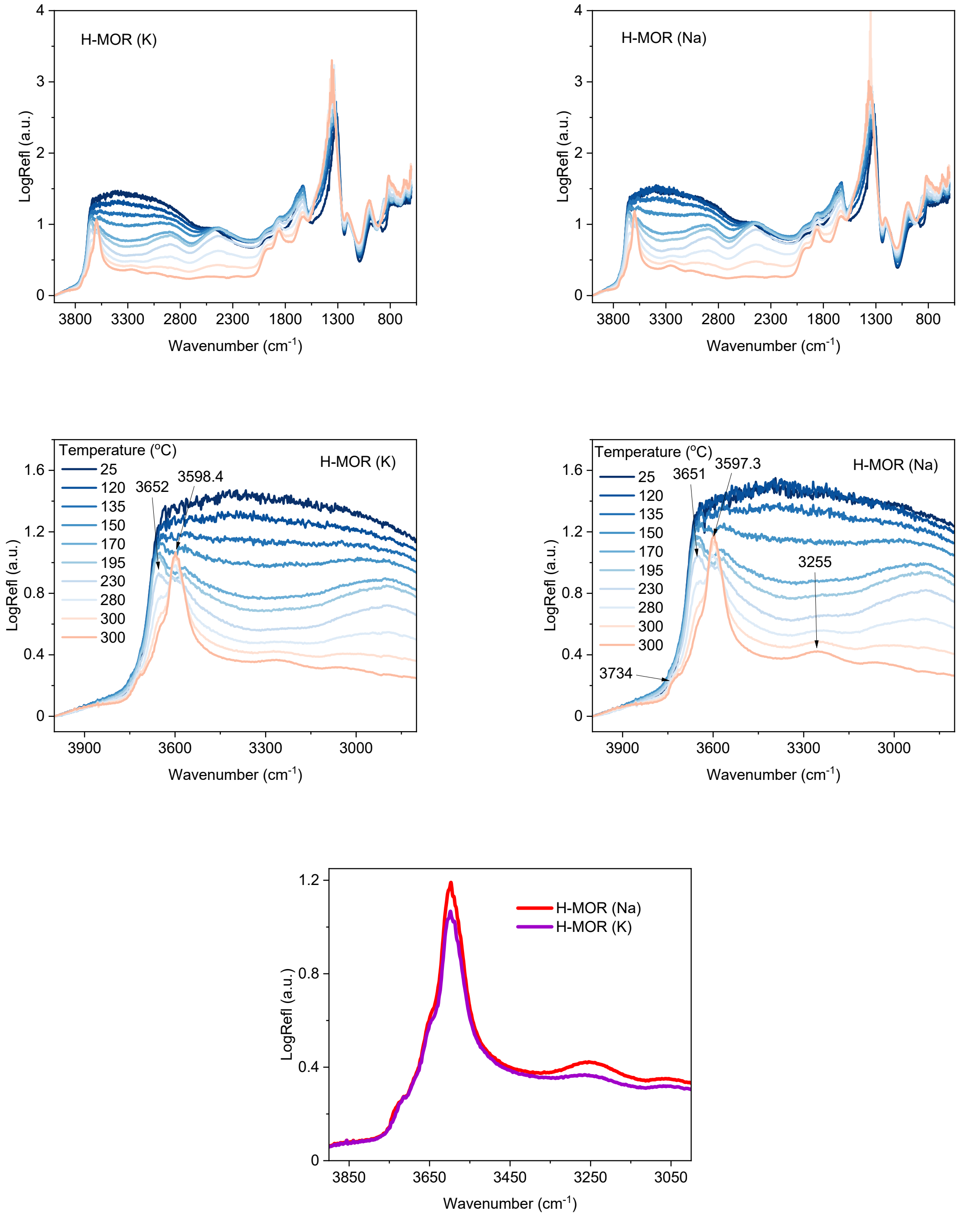

Figure S12. VT-DRIFS experiments of the protonic form H-MOR ion exchanged from Na-MOR and K-MOR respectively. Last panel shows the comparison in the region of interest of both samples of the high temperature spectra. The bands at 3255 cm^{-1} correspond to hydrogen-bonded silanol nests.[4]

Acid site characterization

Table S5 NH_3 released during the temperature programmed calcination of the NH_4-Form and the amount of NH_3 released from the subsequent back-titration. Uncertainty ± 5%

Sample	Al_{MP-AES} (µmol/g)	NH_3 NH_4-Form (µmol/g)	NH_3 TPD (µmol/g)
H-MOR (K)	1997	2080	1488
H-MOR (0.5K)	2276	1891	1386
H-MOR (Na)	1971	2101	1296

Table S6. showing the results for the propylamine-TPD sequences. We quantified both propene and NH_3 originating from propylamine. A subsequent TPO step allowed us to quantify the amount of CO_2 released from coke (on a per molecule basis). This allowed us to create a carbon balance. Lastly, we relate the amount of propene released to the amount of Al present (from MP-AES) to define an accessibility index.[5] Uncertainty ± 5%

Sample	Al_{MP-AES} (µmol/g)	$Propene_{TPD}$ (µmol/g)	NH_3 from C_3 (µmol/g)	CO_2 from TPD& TPO (µmol/g)	Carbon balance (%)	Accessibility index (%)
H-MOR (K)	1997	490	952	361	64%	25%
H-MOR (0.75K)	2149	713	983	205	79%	33%
H-MOR (0.5K)	2276	866	997	246	95%	38%
H -MOR (0.25K)	1999	923	1115	217	89%	46%
H-MOR (0.10K)	2077	1114	976	92	117%	54%
H-MOR (Na)	1971	1293	1013	63	130%	66%
H-SSZ-13	2400	1336	1036	35	130%	56%
H-CBV10-Ads	2200	1246	940	49	134%	57%

In addition to the above experiments, we also performed consecutive propylamine-TPD runs on the H-MOR (K) and H-MOR (Na) samples to investigate whether the cation nature had any impact on the structural integrity of the materials (Table S7).[6,7] During the TPD sequence the material is exposed several times to high temperatures (670 °C) in an oxidative atmosphere which results in the loss of some acid sites. Using the NH_3 concentration as a more reliable measure we find that 31% and 35% of acid sites are lost for the H-MOR (K) and H-MOR (Na), respectively. As the loss is comparable for both samples we conclude that the cation speciation in the synthesis did not have an impact on the structural integrity of the framework.

Table S7. Acid site concentration determined from the NH_3 signal released during repeated propylamine TPD. Uncertainty is ± 5%

Sample	NH_3 from C_3 (µmol/g)	Acid site loss
H-MOR (K)	948	30.7%
H-MOR (K) 2x	657	
H-MOR (Na)	1289	34.8%
H-MOR (Na) 2x	840	

Investigation of constrained environment via catalytic test reactions

Propene oligomerization

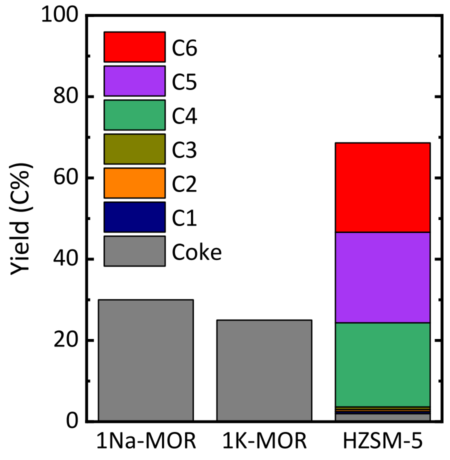

Figure S13. Yield of product obtained after 15 min on stream for H-MOR (Na), H-MOR (K) (Si/Al = ca. 7) and H-ZSM-5 (Si/Al = 50). Coke yield is estimated by the carbon balance compared to the feed and rest to 100 C% correspond to unreacted propene. 230 °C, 2 bar (0.05 bar propene), GHSV, 60000 $cm^3/g/h$

Isobutane cracking

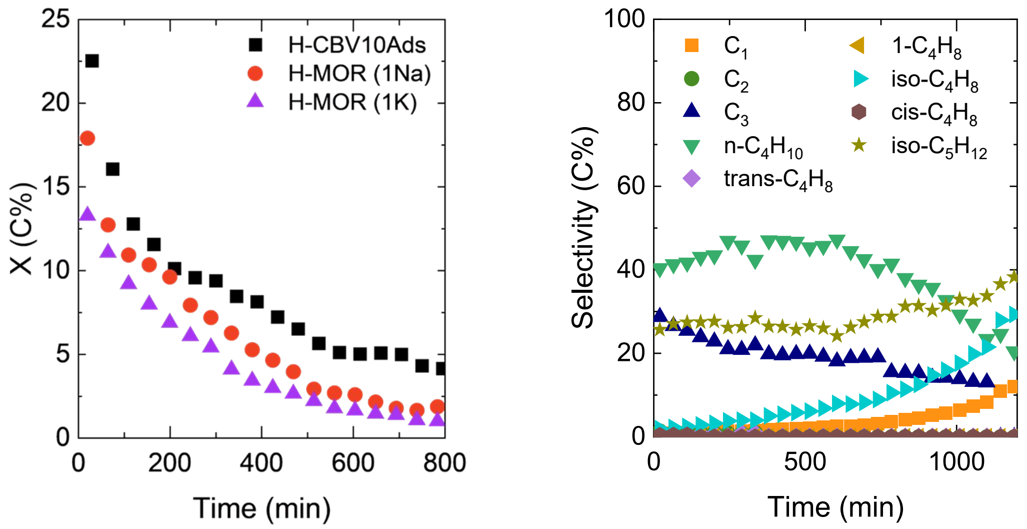

Figure S14. (Left) Conversion of isobutane (iso-C_4H_{10}) as a function of the time on stream for the commercial and in-house synthesized MOR crystals in their H-form. (Right) Product distribution with time obtained with the H-MOR (K), similar product distribution obtained for the three catalysts. 450 °C, 1 bar (0.15 bar isobutane), GHSV, 30000 $cm^3/g/h$

References

1 C. H. Chi and L. B. Sand, *Zeolites*, 1985, **5**, 309–312.

2 S. Prodinger and M. A. Derewinski, in *Nanoporous Materials for Molecule Separation and Conversion*, eds. J. Liu and F. Ding, Elsevier, Amsterdam, 2020, pp. 65–88.

3 A. Katerinopoulou, T. Balic-Zunic and L. F. Lundegaard, *J. Appl. Crystallogr.*, 2012, **45**, 22–27.

4 S. Prodinger, M. A. Derewinski, A. Vjunov, S. D. Burton, I. Arslan and J. A. Lercher, *J. Am. Chem. Soc.*, 2016, **138**, 4408–4415.

5 S. Prodinger, K. Kvande, B. Arstad, E. Borfecchia, P. Beato and S. Svelle, *ACS Catal.*, 2022, **12**, 2166–2177.

6 S. Prodinger, M. A. Derewinski, Y. Wang, N. M. Washton, E. D. Walter, J. Szanyi, F. Gao, Y. Wang and C. H. F. Peden, *Appl. Catal. B Environ.*, 2017, **201**, 461–469.

7 F. Gao, Y. Wang, N. M. Washton, M. Kollár, J. Szanyi and C. H. F. Peden, *ACS Catal.*, 2015, **5**, 6780–6791.
